# Sugammadex is effective in reversing rocuronium in the presence of antibiotics

**DOI:** 10.1186/1471-2253-14-69

**Published:** 2014-08-15

**Authors:** Mark E Hudson, Henk Rietbergen, Jacques E Chelly

**Affiliations:** 1 Department of Anesthesiology, University of Pittsburgh, Pittsburgh, PA, USA; 2 MSD, Oss, The Netherlands

**Keywords:** Sugammadex, Rocuronium, Neuromuscular blockade, Antibiotics

## Abstract

**Background:**

The effectiveness of sugammadex in reversing rocuronium-induced neuromuscular blockade (NMB) in the presence of drugs that may potentiate NMB remains to be fully established. The aim of this *post*-*hoc* analysis of data from a Phase III clinical trial (VISTA; NCT00298831) was to investigate the impact of antibiotics on recovery from rocuronium-induced NMB after administration of sugammadex for reversal, and compared the neuromuscular recovery in patients who received antibiotics preoperatively with those who did not.

**Methods:**

A Phase III, multicenter, open-label study designed to reflect potential use of sugammadex in clinical practice was conducted at 19 sites. Data obtained from patients who received antibiotics were compared with the cohort of patients who underwent the same protocol without antibiotics. Each subject received rocuronium 0.6 mg/kg for muscle relaxation, after which tracheal intubation was performed; patients were also permitted to receive maintenance doses of rocuronium 0.15 mg/kg to maintain the desired level of NMB throughout the operation, as required.. At least 15 min after the last rocuronium dose, patients received sugammadex 4.0 mg/kg for reversal. Neuromuscular monitoring was continued until a train-of-four (TOF) ratio of ≥0.9 was achieved or the anesthetic was discontinued.

**Results:**

The presence of antibiotics prior to the administration of sugammadex did not affect the recovery time from rocuronium-induced NMB when sugammadex 4.0 mg/kg was administered at least 15 min after the last dose of rocuronium. In the presence of antibiotics, the geometric mean (95% CI) time from administration of sugammadex 4.0 mg/kg to recovery of the TOF ratio to ≥0.9 was 1.6 (1.4–1.9) min (range: 0.7–10.5 min), compared with 2.0 (1.8–2.3) min (range: 0.7–22.3 min) for patients who did not receive antibiotics.

**Conclusions:**

These findings suggest that prophylactic antibiotic use is unlikely to have a major impact on the recovery time from rocuronium-induced NMB with sugammadex reversal.

**Trial registration:**

ClinicalTrials.gov Identifier: NCT00298831.

## Background

Sugammadex is a modified γ-cyclodextrin that effectively reverses moderate and deep neuromuscular blockade (NMB) induced by the non-depolarizing muscle relaxants rocuronium and vecuronium [1-5]. However, the effectiveness of sugammadex in reversing rocuronium in the presence of drugs that potentiate NMB remains to be fully established. Certain classes of drugs are known to potentiate the activity of the neuromuscular blocking agents (NMBAs) if used in the perioperative setting [6-11], including several antibiotics [6,9-11]. Furthermore, the presence of certain antibiotics may limit the ability of traditional reversal agents such as neostigmine to reverse NMB [6,12]. In cases where neostigmine is found to be ineffective, this may be a consequence of the antibiotic potentiating NMB through inhibition of acetylcholine release from the nerve terminal. A reduced concentration of acetylcholine would reduce competition for the NMBA to bind to the nicotinic acetylcholine receptor , thereby potentiating the NMB. However, since neostigmine works by reducing enzymatic breakdown of acetylcholine in the neuromuscular junction to increase the concentration of acetylcholine, thereby shifting the competition between acetylcholine and the NMBA for the binding site in favor of acetylcholine, a reduced availability of acetylcholine may subsequently limit the effectiveness of neostigmine.

Since sugammadex works by a different mechanism to neostigmine, forming 1:1 complexes with rocuronium or vecuronium, encapsulating the NMBA, resulting in its inactivation [13], it is not anticipated that antibiotics will impact on its ability to reverse rocuronium- or vecuronium-induced NMB. In the current study, we investigate this further.

Since in previous studies conducted to assess the safety and efficacy of sugammadex, patients who received antibiotics perioperatively were excluded, a *post*-*hoc* analysis of data from a Phase III clinical trial (study ID: NCT00298831) was performed to assess the effects of antibiotic administration on sugammadex properties in reversing NMB produced by rocuronium. In an attempt to reflect clinical practice in this Phase III study, there were no restrictions on anesthetic regimen or procedure, with only limited restrictions on concomitant medications. However, medications in a dose and/or at a time point known to interfere with the action of non-depolarizing NMBAs, including antibiotics, were listed among the exclusion criteria. Despite this, a relatively large number of patients received prophylactic antibiotics (per the routine practice for several study sites) in deviation from the study protocol, thereby allowing comparison with those patients who did not receive antibiotics.

## Methods

The Phase III (VISTA) study was a multicenter, open-label study conducted at 19 sites in the USA between October 2005 and May 2006, data from which have been published previously [1], and was designed to reflect a potential use of sugammadex in clinical practice. This study was conducted in accordance with principles of Good Clinical Practice and was approved by the appropriate institutional review boards and regulatory agencies (Additional file 1). Written informed consent was obtained from all patients. Antibiotic use was listed under the exclusion criteria, as antibiotics may interfere with the action of non-depolarizing NMBAs. In the current *post*-*hoc* analysis to establish the impact of antibiotic administration on the ability of sugammadex to reverse the muscle relaxant property of rocuronium, data obtained from sugammadex-treated patients who received antibiotics (deviating from the study protocol) were compared with the cohort of patients who underwent the same protocol without antibiotics.

As previously described [1], inclusion criteria included age between 18–70 years with American Society of Anesthesiologists physical status I–III and scheduled to undergo elective surgery in the supine position under general anesthesia requiring muscle relaxation. Non-standardized anesthesia was induced and maintained with an intravenous opioid, an anesthetic, and other agent(s) according to the clinical need of each subject. Anesthesia practices not specified in the protocol were to be consistent with the routine practices at the study site. Most patients (99%) included in the study received propofol and/or an opioid for induction of anesthesia; the remaining patients received either sevoflurane or desflurane. The most common anesthetics used for the maintenance of anesthesia were sevoflurane and desflurane, although some patients received either propofol or isoflurane.

Neuromuscular monitoring was performed continuously at the adductor pollicis muscle with acceleromyography (TOF-Watch® SX; Organon Ireland Ltd, a division of Merck and Co., Dublin, Ireland). After calibration of the TOF-Watch, each subject received rocuronium 0.6 mg/kg for muscle relaxation, after which tracheal intubation was performed. Patients were permitted to receive maintenance doses of rocuronium 0.15 mg/kg upon reappearance of the second twitch of the train-of-four (TOF) to maintain NMB throughout the operation as required. At least 15 min after the last dose of rocuronium, patients received sugammadex 4.0 mg/kg for reversal. Neuromuscular monitoring was continued until a TOF ratio of ≥0.9 was achieved or the anesthetic was discontinued. The primary efficacy variable was the time from the start of sugammadex administration until recovery of the TOF ratio to ≥0.9.

### Data analysis

Recovery times were summarized by geometric mean (95% confidence interval [CI]) times, as these times were expected to follow a skewed distribution and the geometric mean is robust against data distributed in this way [14]. Data were also summarized as number and percentage of patients recovering to a TOF ratio of ≥0.9 within each minute time interval after sugammadex administration. An analysis of variance (ANOVA) model was used to investigate the effect of depth of NMB at the time of sugammadex administration (number of twitches to TOF stimulation: 0 or ≥1) and use of antibiotics on the (log) time to recovery to a TOF ratio of ≥0.9. This ANOVA model also included study center.

Safety was assessed by adverse events (AEs, coded by Medical Dictionary for Regulatory Activities version 9.1), laboratory variables, vital signs, and clinical signs of neuromuscular recovery.

## Results

Of the 224 patients enrolled in the original trial, only patients who received sugammadex were included in the current analysis (*n* = 197). Of these 197 patients, 64 received an antibiotic known to interfere with NMBAs before recording any efficacy parameters, including kanamycin (*n* = 26), gentamicin (*n* = 20), vancomycin (*n* = 9), clindamycin (*n* = 6) and bacitracin (*n* = 3). Table 1 presents the demographic data for the two groups. The route of administration of antibiotics was intravenous (*n* = 34); oral (*n* = 1); intra-articular (*n* = 1) or in the abdominal wash (*n* = 28) (Table 2).

**Table 1:** Baseline demographics of patients receiving sugammadex in the overall study

	**Antibiotics ( *n * = 64)**	**No antibiotics ( *n * = 133)**
Age, years, mean (SD)	57 (13)	51 (16)
Weight, kg, mean (SD)	87 (20)	82 (20)
Height, cm, mean (SD)	172 (9)	169 (10)
Gender, *n* (%)		
Male	39 (61)	55 (41)
Race, *n* (%)		
Asian	1 (2)	4 (3)
Black, of African heritage	5 (8)	13 (10)
White/Caucasian	58 (91)	114 (86)
Other	0 (0)	2 (2)
ASA class, *n* (%)		
I	4 (6)	23 (17)
II	51 (80)	84 (63)
III	9 (14)	26 (20)

*ASA*: American Society of Anesthesiologists; *SD*: standard deviation.

**Table 2:** Frequency of antibiotics by route of administration

	**Route of administration**				
	**Intravenous**	**Oral**	**Intra-articular**	**Abdominal wash**	**Total**
Kanamycin	0	0	0	26	26
Gentamycin	19	1	0	0	20
Vancomycin	9	0	0	0	9
Clindamycin	6	0	0	0	6
Bacitracin	0	0	1	2	3
	34	1	1	28	64

Four patients who received antibiotics and 16 who did not receive antibiotics were excluded from the current efficacy analysis because they did not have data available for a time to the TOF ratio of 0.9 (data missing or deemed unreliable by a central independent adjudication committee; reasons for missing or unreliable data included technical difficulties with the monitoring equipment and a clinical need to discontinue monitoring prior to reaching a TOF ratio of 0.9). In the presence of antibiotics (*n* = 60), the geometric mean (95% CI) time from administration of sugammadex 4.0 mg/kg to recovery of the TOF ratio to ≥0.9 was 1.6 (1.4–1.9) min (range: 0.7–10.5 min), compared with 2.0 (1.8–2.3) min (range: 0.7–22.3 min) for patients who did not receive antibiotics (*n* = 117).

In the original study, which was designed at the time to reflect a potential use in clinical practice, reversal at ≥15 min after the last dose of rocuronium resulted in a variability in the depth of NMB at the time of reversal [1]. As a consequence, while some patients in the study may have received sugammadex 4.0 mg/kg at an appropriate level of NMB, some have received sugammadex at a deeper NMB than recommended for this dose. Recovery times by antibiotic administration and NMB depth for patients with data available for the number of twitches at the start of sugammadex administration are shown in Table 3; analyses of the recovery times showed a difference in recovery between patients with zero twitches and those with one or more twitches (*P* < 0.0001), but no statistically significant difference was observed between patients who received antibiotics and those who did not, *P* = 0.30. The estimated treatment effect (adjusted for center effects) for the ratio of the geometric means for patients who received no antibiotics and those who did was 1.11 (95% CI 0.91–1.35), while for patients who had zero twitches and those who had ≥1 twitch, the ratio of the geometric means was 1.46 (95% CI 1.23–1.73).

**Table 3:** **Summary of times to recovery of the TOF ratio to 0.9 following administration of sugammadex** ≥**15 min after the last dose of rocuronium** (**analyzed by antibiotic administration and NMB depth for patients with data available for the number of twitches at the start of sugammadex administration**)

**Antibiotics**	**No. of twitches**	***n***	**Geometric mean recovery time (min)**	**95% confidence interval**
No	0	60	2.4	2.0–2.7
No	≥1	51	1.6	1.4–1.9
**No**		**111**^†^	**2.0**	**1.8**–**2.2**
Yes	0	24	2.2	1.7–2.8
Yes	≥1	33	1.3	1.2–1.5
**Yes**		**57**^†^	**1.6**	**1.4**–**1.9**

*NMB*: neuromuscular blockade; *TOF*: train-of-four.

^†^Nine patients (three patients who received antibiotics and six who did not) had data available for recovery to a TOF ratio of ≥0.9, but no data regarding the depth of NMB (number of twitches) at the time of sugammadex administration.

The text in bold indicates the sub-totals of antibiotics or no antibiotics, irrespective of the NMB depth.

**Figure 1:**
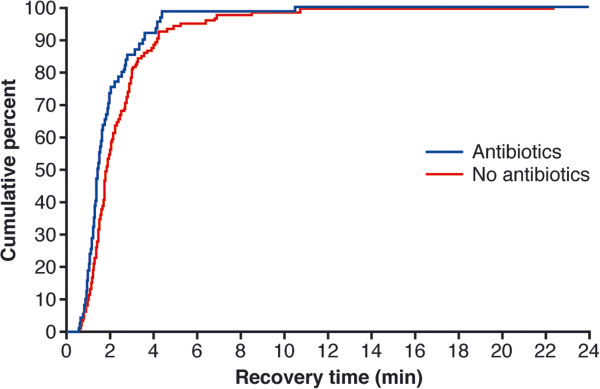
Cumulative percentage of patients who recovered, after sugammadex administration, to TOF ratio ≥0.9, by receiving antibiotics or not (patients with available recovery data).

Table 4 presents, per minute, the cumulative number and percentage of patients who recovered after sugammadex administration to TOF ratio ≥0.9. For one patient (1.7%) who received antibiotics, the time from sugammadex administration to recovery of the TOF ratio ≥0.9 took more than 5 min (10.5 min), compared with seven patients (6.0%) who did not receive antibiotics (Table 4; Figure 1). For the patient in the antiobiotic group with the relatively long recovery time to a TOF ratio of ≥0.9 (10.5 min), there were zero twitches in response to TOF stimulation at the time of sugammadex administration, potentially meaning that the depth of NMB was deeper than recommended for the dose of sugammadex used, which may help to explain the slower recovery observed. Similarly, three of the patients with recovery times >5 min in the group that did not receive antibiotics had zero twitches at the time of sugammadex administration (recovery times were 6.9, 6.9 and 22.3 min), while one patient had no data available for the depth of NMB (recovery time 10.7 min); the remaining three patients had ≥1 twitches (recovery times 5.2, 6.4 and 8.5 min).

**Table 4:** **Cumulative number and percentage of patients who recovered after sugammadex administration to TOF ratio** ≥**0.9**, **by receiving antibiotics or not** (**patients with available recovery data**)

**Time to TOF ratio** ≥**0.9**	**Antibiotics ( *n * = 60)**		**No antibiotics ( *n * = 117)**	
	***n***	**%**	***n***	**%**
≤1 min	9	15.0	11	9.4
≤2 min	44	73.3	63	53.9
≤3 min	51	85.0	94	80.3
≤4 min	55	91.7	103	88.0
≤5 min	59	98.3	110	94.0

*TOF*: train-of-four.

Overall, the majority of patients who received sugammadex reported at least one AE: 96.9% of patients who received an antibiotic and 97.7% of patients who did not receive an antibiotic. Of patients who received antibiotics, four (6.3%) experienced AEs considered possibly related to sugammadex: procedural hypertension (*n* = 1), procedural hypotension (*n* = 1), wheezing (*n* = 1) and increased blood creatinine phosphokinase (*n* = 1); 18 (13.5%) patients who did not receive antibiotics reported an AE considered by the investigator to be possibly related to sugammadex, with the most common being hematuria (*n* = 4). There were eight serious AEs in the study as a whole, two in patients receiving an antibiotic and six in patients not receiving an antibiotic; none of these serious AEs were considered related to sugammadex.

## Discussion and Conclusions

In the current *post*-*hoc* analysis, the presence of antibiotics prior to the administration of sugammadex 4.0 mg/kg did not affect the ability of sugammadex to reverse the NMB produced by rocuronium when administered at least 15 min after the last dose of rocuronium, with no significant difference observed in recovery time between patients who received preoperative antibiotics compared with a cohort of patients who underwent the same protocol without antibiotic administration. While it cannot be concluded from these analyses that antibiotics have no effect on sugammadex reversal of rocuronium-induced NMB, the data presented suggest that prophylactic antibiotic use is unlikely to have a major impact on recovery time.

## Competing interests

Mark Hudson was the principal site investigator for the original study; his institution received funding from Merck for the conduct of this study. Henk Rietbergen is an employee of MSD, who may own stock and/or hold stock options in the company. Jacques Chelly reports no conflicts of interest.

## Authors’ contributions

MEH was involved in the design and conduct of the original study, data collection, and prepared the first draft of this brief report. HR performed the data analyses for this post-hoc analysis, critically reviewed drafts of the report, and is the archival author responsible for maintaining study records. JEC was involved in the conduct of the original study, data collection, and critically reviewed drafts of the report. All authors read and approved the final manuscript.

## Authors’ information

MEH is an Associate Professor, Department of Anesthesiology, University of Pittsburgh. HR is a Prinicipal Statistician, MSD, Oss, The Netherlands. JEC is a Professor of Anesthesiology and Orthopedic Surgery, University of Pittsburgh.

## Pre-publication history

The pre-publication history for this paper can be accessed here:

http://www.biomedcentral.com/1471-2253/14/69/prepub

## Supplementary Material

Additional file 1 List of sites and Institutional Review Boards for the VISTA study. Click here for file
