# Supplementary material for: Sugammadex is effective in reversing rocuronium in the presence of antibiotics
Source: BMC Anesthesiol. 2014 Aug 15;14:69. doi: 10.1186/1471-2253-14-69 (PMC4142447; doi:10.1186/1471-2253-14-69)
Supplement: Additional file 1 — List of sites and Institutional Review Boards for the VISTA study. [file 1471-2253-14-69-S1.docx]

# Additional File 1: List of Sites and Institutional Review Boards for the VISTA study

| Site no. | Site Location | Institutional Review Board |
| --- | --- | --- |
| 101 | Memorial Hermann-Memorial City Hospital, Houston, TX, USA | CPHS/AWC Office, University Center Tower, Houston, TX, USA |
| 102 | University of Texas Southwestern Medical Center, Dallas, TX, USA | Institutional Review Board, The University of Texas Southwestern Medical Center at Dallas, Dallas, TX, USA |
| 103 | UCLA Medical Center, Los Angeles, CA, USA | Office for Protection of Research Subjects (OPRS), Los Angeles, CA, USA |
| 104 | Hospital of the University of Pennsylvania, Philadelphia, PA, USA | University of Pennsylvania, Office of Regulatory Affairs, Philadelphia, PA, USA |
| 107 | Wishard Memorial Hospital, Indianapolis, IN, USA | Indiana University Purdue University Indianapolis, Research Sponsored Programs, Indianapolis, IN, USA |
| 108 | Boston Medical Center, Boston, MA, USA | Western Institutional Review Board, Olympia, WA, USA |
| 112 | Harborview Medical Center, Seattle, WA, USA | Western Institutional Review Board, Olympia, WA, USA |
| 114 | Thomas Jefferson University, Philadelphia, PA, USA | Institutional Review Board, Thomas Jefferson University, Office of Scientific Affairs, Philadelphia, PA, USA |
| 115 | UPMC-Shadyside Hospital, Pittsburgh, PA, USA | Western Institutional Review Board, Olympia, WA, USA |
| 116 | University of Arkansas for Medical Sciences, Little Rock, AR, USA | University of Arkansas for Medical Sciences, Institutional Review Board, Little Rock, AR, USA |
| 117 | University of Virginia Health System, Charlottesville, VA, USA | University of Virginia, Human Investigation Committee, Charlottesville, VA, USA |
| 118 | University of Rochester, Rochester, NY, USA | Western Institutional Review Board, Olympia, WA, USA |
| 119 | Mayo Clinic, Rochester, MN, USA | Mayo Foundation Institutional Review Board, Rochester, MN, USA |
| 120 | University of Maryland School of Medicine, Baltimore, MD, USA | Human Protection Research Office/IRB, University of Maryland at Baltimore, Baltimore, MD, USA |
| 121 | LAC + USC Medical Center, Los Angeles, CA, USA | University of Southern California Health Science Campus Institutional Review Board, LAC + USC Medical Center, Los Angeles, CA, USA |
| 122 | Magee Women’s Hospital, Pittsburgh, PA, USA | Western Institutional Review Board, Olympia, WA, USA |
| 123 | Loma Linda University School of Medicine, Loma Linda, CA, USA | Loma Linda University Institutional Review Board, Loma Linda, CA, USA |
| 124 | Long Beach Memorial Medical Center, Long Beach, CA, USA | Memorial Health Services Office of Research Administration, Long Beach Medical Center, Long Beach, CA, USA |
| 125 | Vanderbilt University Medical Center, Nashville, TN, USA | Vanderbilt University Institutional Review Board, Nashville, TN, USA |
